# Genetic and metabolic drivers of membrane remodeling in *Clostridium thermocellum* under alcohol stress

**DOI:** 10.1128/msystems.01345-25

**Published:** 2026-03-05

**Authors:** Eashant Thusoo, Tyler Jacobson, Bishal D. Sharma, Isabella M. Colón, Lee R. Lynd, Daniel G. Olson, Daniel Amador-Noguez

**Affiliations:** 1 Center for Bioenergy Innovation, Oak Ridge National Laboratory 6146, Oak Ridge, Tennessee, USA; 2 Department of Bacteriology, University of Wisconsin–Madison 205263, Madison, Wisconsin, USA; 3 Thayer School of Engineering, Dartmouth College 145792, Hanover, New Hampshire, USA; 4 Great Lakes Bioenergy Research Center, University of Wisconsin-Madison 5228, Madison, Wisconsin, USA; Korea Advanced Institute of Science and Technology, Yuseong-gu, Daejeon, Republic of Korea

**Keywords:** *Clostridium thermocellum*, *Acetivibrio thermocellus*, branched-chain fatty acid synthesis, lipid membrane remodeling, mass spectrometry

## Abstract

*Clostridium thermocellum* is a leading candidate for consolidated bioprocessing of lignocellulosic biomass into biofuels due to its native cellulolytic capabilities. Beyond ethanol, *C. thermocellum* is being developed as a platform for producing higher-chain alcohols such as isobutanol and *n*-butanol. However, its physiological adaptations to alcohol stress remain poorly understood. Here, we investigate how *C. thermocellum* remodels its membrane lipid composition in response to exogenous ethanol, *n*-butanol, isobutanol, and butyrate. Exposure to linear alcohols such as *n*-butanol or to organic acids like butyrate increased the proportion of straight-chain fatty acids in the membrane at the expense of branched-chain species, whereas exposure to the branched alcohol isobutanol produced the opposite effect. Isotope tracer experiments demonstrated that *C. thermocellum* directly incorporates the carbon backbones of exogenous alcohols and acids into fatty acids, providing a mechanistic basis for these contrasting shifts. We show that the bifunctional aldehyde/alcohol dehydrogenase AdhE is essential for the assimilation of exogenous alcohols into fatty acids, acting through its oxidative activity by first oxidizing alcohols to aldehydes and then converting them to acyl-CoA intermediates. Deletion of the pyruvate:ferredoxin oxidoreductase isozyme pfor4 abolished branched-chain fatty acid synthesis, but supplementation with isobutanol restored production, indicating that Pfor4 substitutes for the canonical branched-chain α-keto acid dehydrogenase complex. These findings reveal two distinct routes for branched-chain fatty acid production in *C. thermocellum*: a Pfor4-dependent pathway from α-keto acid intermediates derived from amino acid synthesis, and an AdhE-dependent salvage pathway that assimilates exogenous branched-chain alcohols.

**IMPORTANCE:**

This study identifies key mechanisms of *Clostridium thermocellum* membrane remodeling under alcohol stress, showing that AdhE mediates incorporation of exogenous alcohols into fatty acids, while Pfor4 drives branched-chain fatty acid synthesis in the absence of the canonical Bkd complex. These findings highlight actionable targets for metabolic engineering to enhance solvent tolerance and improve the robustness and productivity of *C. thermocellum* as a biofuel-producing platform.

## INTRODUCTION

*Clostridium thermocellum* (also referred to as *Acetivibrio thermocellus*) (1) is a thermophilic, obligate anaerobe with a native ability to deconstruct and metabolize lignocellulosic biomass. Its ability to degrade crystalline cellulose and channel the resulting sugars into fermentation pathways makes it a strong candidate for consolidated bioprocessing, an approach that integrates biomass deconstruction and biofuel production into a single organism. Considerable metabolic engineering efforts have been directed at improving ethanol production in *C. thermocellum*, resulting in strains capable of achieving high yields, over 80% of the theoretical maximum in some cases. Despite these advances, ethanol titers remain a key limitation: current strains typically reach only 25–30 g/L, falling short of the 40–50 g/L range generally considered necessary for economically viable, large-scale biofuel production (2–4).

Beyond optimizing ethanol yields, research has focused on expanding the metabolic capacity of *C. thermocellum* to synthesize higher-chain alcohols like isobutanol and *n*-butanol (5–9). While isobutanol is a native, but minor, fermentation product of *C. thermocellum*, metabolic engineering has successfully increased its production to approximately 5 g/L (5). In contrast, *n*-butanol is not naturally produced by *C. thermocellum*, and efforts to introduce heterologous biosynthetic pathways have yielded limited success, with reported titers reaching only 195 mg/L (6).

*C. thermocellum* employs a modified Embden–Meyerhof–Parnas (EMP) glycolytic pathway in which several phosphorylation steps utilize pyrophosphate (PPi) rather than ATP. This PPi-dependent glycolysis operates with a markedly lower overall thermodynamic driving force compared to the highly exergonic Entner–Doudoroff (ED) pathway of the industrially relevant *Zymomonas mobilis* and is also less favorable than the canonical ATP-dependent EMP pathway found in model organisms such as *Escherichia coli* (4, 7–10). Although these unique metabolic features likely support *C. thermocellum*’s survival in its native niche, they are generally considered disadvantageous for engineering strains optimized for biofuel production. As ethanol accumulates during fermentation, the thermodynamic driving force of glycolysis and ethanol production is thought to diminish, approaching equilibrium, which ultimately halts sugar catabolism. This thermodynamic limitation has been proposed as a key factor constraining ethanol titers in *C. thermocellum* (4, 8, 11–13). Recent metabolic engineering approaches to improve the thermodynamics of its glycolytic pathway, such as switching the cofactor specificity of glycolytic enzymes such as phosphofructokinase from PPi to ATP hydrolysis, have resulted in increased ethanol titers (4).

In addition to the limited thermodynamic driving force of glycolytic and fermentative pathways, another likely barrier to achieving higher ethanol titers in *C. thermocellum* is its limited tolerance to high ethanol concentrations (14, 15). In many microorganisms, membrane remodeling plays a central role in mitigating alcohol-induced stress. For instance, *Zymomonas mobilis* enhances its ethanol tolerance by increasing the abundance of cyclopropane fatty acids (19:Cyclo) in its membrane, a modification thought to reduce membrane permeability and limit ethanol influx (16). Similarly, other bacteria adapt to solvent stress by altering fatty acid chain length, saturation levels, or branching patterns. For example, *Clostridium acetobutylicum* increases the proportion of saturated fatty acids under furans, phenol, and weak acid stress to reduce membrane fluidity, while *Escherichia coli* increases cyclopropane fatty acid content during the stationary phase or environmental stress (17–19). These adaptive changes help maintain membrane integrity, protecting cellular functions in the presence of toxic solvents.

Despite its industrial relevance, the molecular mechanisms underlying alcohol tolerance in *C. thermocellum* remain poorly understood. To date, most studies have focused on global metabolomic, transcriptomic, and proteomic responses to ethanol stress (20), while analyses of membrane composition have primarily focused on differences between wild-type strains and ethanol-tolerant strains (21, 22). Notably, these studies have focused exclusively on ethanol, with no characterization of *C. thermocellum*’s response to higher molecular weight alcohols such as isobutanol or *n*-butanol. The extent to which *C. thermocellum’*s membrane composition is modulated in response to ethanol and related stressors is not well characterized. This represents a significant barrier to the rational engineering of strains with enhanced solvent resistance.

To address this knowledge gap, we combined stable isotope tracer experiments with liquid chromatography-mass spectrometry (LC-MS) lipidomics to investigate how wild-type and engineered *C. thermocellum* strains remodel their membrane composition in response to exogenous alcohols and organic acids. This approach revealed previously uncharacterized membrane remodeling responses and provided new mechanistic insights into the processes driving these alterations.

## RESULTS

### Experimental design

To investigate membrane remodeling under solvent-induced stress, wild-type and engineered strains of *C. thermocellum* were inoculated anaerobically at a starting OD_600_ of 0.05 into thermophilic clostridia media (MTC), a minimal medium containing cellobiose as a carbon source and supplemented with *n*-butanol (1.5 g/L), isobutanol (3 g/L), ethanol (5 g/L), or butyrate (1.5 g/L). These compounds were chosen for their relevance to industrial fermentation and the emerging bioeconomy. *n*-butanol, isobutanol, ethanol, and butyrate concentrations were determined from preliminary growth experiments and selected to minimize growth inhibition while eliciting measurable changes in membrane lipid remodeling (Materials and Methods and Table S15). Cultures were harvested at the mid-exponential phase (OD_600_ of 0.45) for lipidomic and metabolomic analyses (Materials and Methods).

### Membrane lipid composition of wild-type *C. thermocellum* is dominated by iso-branched fatty acids.

**Fig 1:**
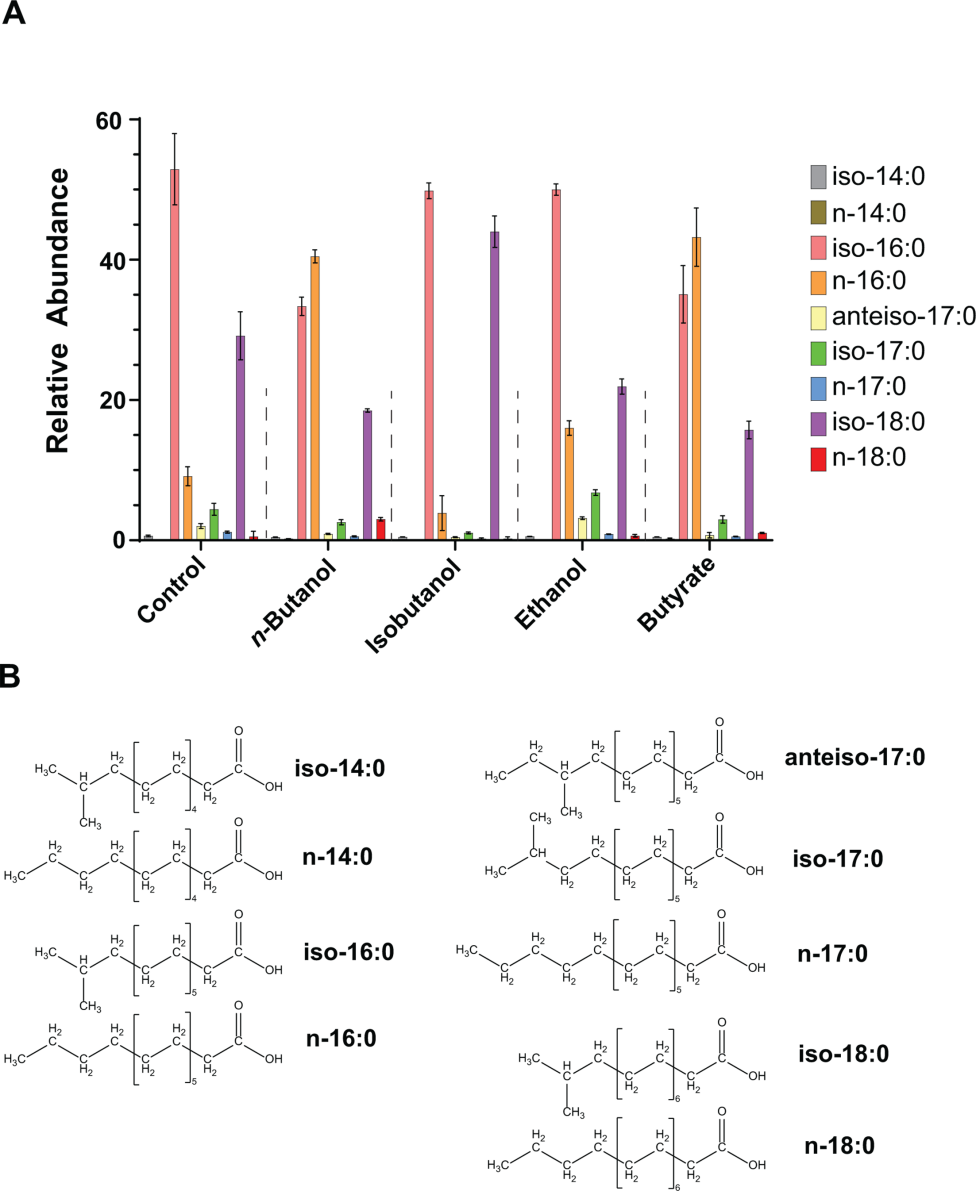
Exposure to exogenous alcohols and organic acids alters the fatty acid composition of *C. thermocellum*. (**A**) Wild-type *C. thermocellum* (DSM1313) was grown in thermophilic clostridia media (MTC) containing cellobiose as the sole carbon source and supplemented with *n*-butanol (1.5 g/L), isobutanol (3 g/L), ethanol (5 g/L), or butyrate (1.5 g/L). Bars show the relative abundance of individual fatty acids within the total fatty acid pool under the indicated exposure conditions. Values represent the average of three biological replicates, with error bars indicating standard deviation. n-Butanol and butyrate increase the relative abundance of straight-chain fatty acids, while isobutanol elevates branched-chain fatty acid levels. Ethanol produces a modest increase in straight-chain fatty acids, less pronounced than that observed with butanol or butyrate. Statistical significance for changes in relative abundance for each fatty acid (two-tailed t-test, supplemented vs. unsupplemented control) is reported in Table S1. (**B**) Chemical structures of identified fatty acids in *C. thermocellum*. Nomenclature: myristic acid (n-14:0), 12-methyltridecanoic acid (iso-14:0), palmitic acid (n-16:0), isopalmitic acid (iso-16:0), heptadecanoic acid (n-17:0), 15-methylpalmitic acid (iso-17:0), 14-methylhexadecanoic acid (anteiso-17:0), stearic acid (n-18:0), and isostearic acid (iso-18:0).

We used LC-MS/MS-based lipidomics to characterize the fatty acid and lipid composition of *C. thermocellum* membranes when grown on minimal media. Analysis of saponified membrane lipids identified the following core set of fatty acids: myristic acid (n-14:0), 12-methyltridecanoic acid (iso-14:0), palmitic acid (n-16:0), isopalmitic acid (iso-16:0), heptadecanoic acid (n-17:0), 15-methylpalmitic acid (iso-17:0), 14-methylhexadecanoic acid (anteiso-17:0), stearic acid (n-18:0), and isostearic acid (iso-18:0). The membrane composition of wild-type *C. thermocellum* is dominated by iso-branched fatty acids, with iso-16:0 and iso-18:0 comprising 52.9% and 29.2% of membrane fatty acids, respectively (Fig. 1A; Table S1).

### Alcohol exposure alters iso-branched to straight-chain fatty acid ratios

Exposure to *n*-butanol, isobutanol, ethanol, or butyrate led to distinct and significant changes in the membrane fatty acid composition of *C. thermocellum* (Fig. 1A). *n*-Butanol exposure triggered a pronounced shift from iso-branched to straight-chain (normal) fatty acids: iso-16:0 and iso-18:0 levels decreased from 52.9% and 29.2% to 33.4% and 18.5%, respectively; while n-16:0 and n-18:0 increased from 9.1% and 0.5% to 40.5% and 3%. Butyrate exposure elicited a nearly identical response, promoting an enrichment in straight-chain fatty acids at the expense of iso-branched species.

In contrast, isobutanol exposure produced the opposite effect, increasing the abundance of iso-branched fatty acids—particularly iso-18:0, which increased from 29.2% to 44%—while reducing the levels of n-16:0 and n-18:0. Ethanol exposure elicited a comparatively milder effect, resulting in moderate decreases in iso-18:0 accompanied by a compensating increase in n-16:0 (Fig. 1A).

### Fatty acid remodeling involves direct assimilation of exogenous alcohols and acids

The opposing shifts in iso-branched versus straight-chain fatty acid composition observed under *n*-butanol and butyrate exposure (which increase straight-chain fatty acids) compared to isobutanol exposure (which increases iso-branched fatty acids) suggest that these exogenous compounds may alter fatty acid composition in *C. thermocellum* by serving as direct precursors in fatty acid biosynthesis. To test this hypothesis, we performed stable isotope tracing experiments using deuterated (^2^H, also referred to as D) and ^13^C-labeled substrates, using each tracer individually: ^2^H_9_-butanol (CD₃–(CD₂)₃–OH), ^2^H₉-isobutanol (CD₃–CD(CD₃)–CD₂–OH), [1-^13^C_1_]butyrate (CH₃–CH₂–CH₂–^13^COOH), and [2-^13^C_1_]ethanol (^13^CH₃–CH₂–OH).

**Fig 2:**
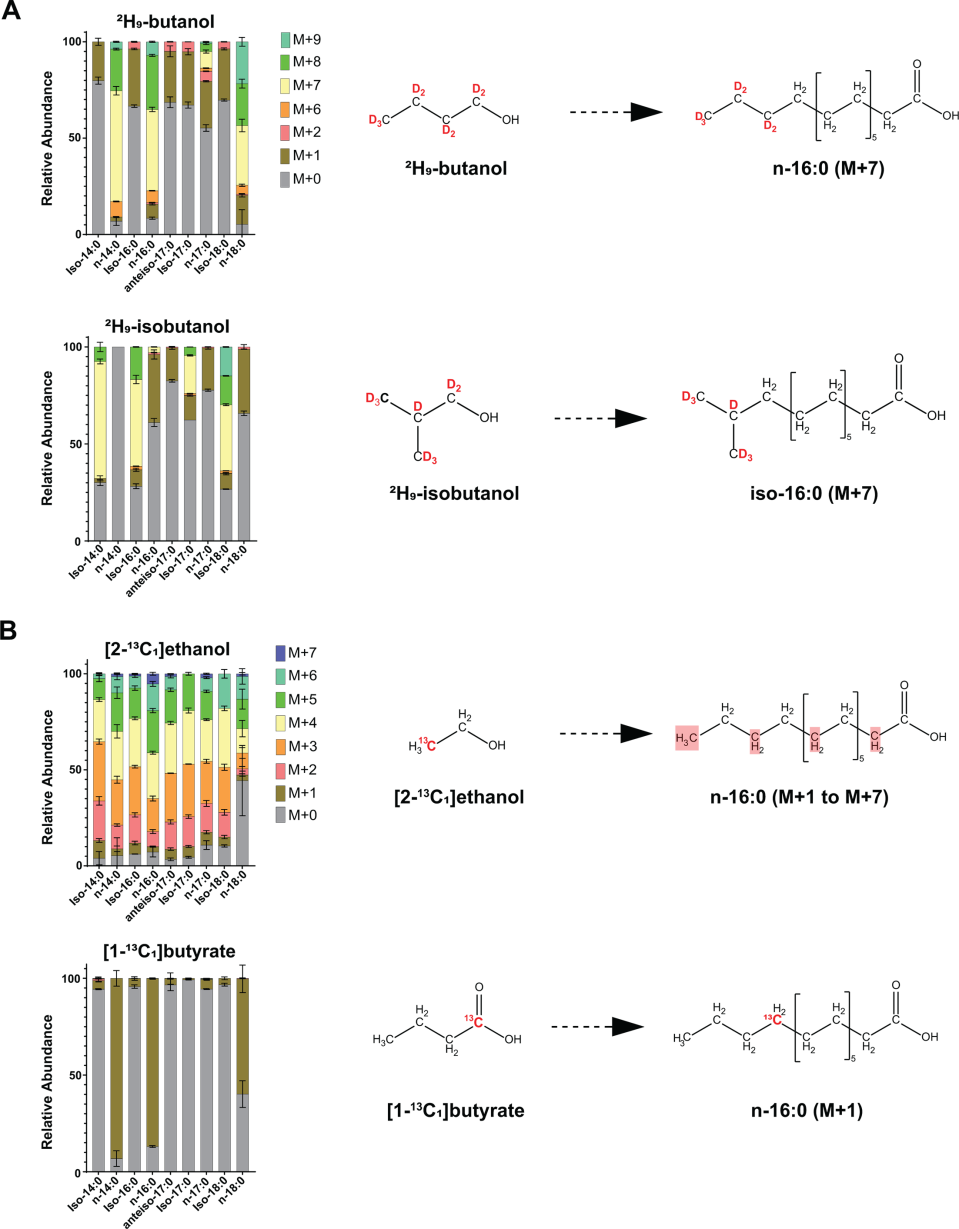
Isotope tracers reveal incorporation of exogenous alcohol and organic acid carbon backbones into fatty acids. Wild-type *C. thermocellum* was grown in minimal media containing cellobiose as the sole carbon source and supplemented with ²H₉-butanol or ²H₉-isobutanol, [1-¹³C_1_]butyrate, or [2-¹³C_1_]ethanol. Bars represent the relative abundance of each isotopologue for the indicated metabolite under each exposure condition. Values are averages of three biological replicates, and error bars indicate standard deviation. Isotopologue distributions for all fatty acids measured are shown in Table S2. (**A**) For ²H₉-butanol and ²H₉-isobutanol, M+0 corresponds to the unlabeled metabolite, M+1 indicates the incorporation of one deuteron, M+2 indicates the incorporation of two deuterons, and so on. A dominant M+7 isotopologue indicates intact incorporation of a single butanol or isobutanol carbon backbone into fatty acids, as shown in the chemical diagrams, using n-16:0 and iso-16:0 as representative examples. M+1 and M+2 species arise from incorporation of exchangeable deuterons from deuterated NADPH, while heavier species (M+8 and M+9) reflect incorporation of both the alcohol carbon backbone and NADPH-derived deuterons. (**B**) For [1-¹³C_1_]butyrate and [2-¹³C_1_]ethanol, M+0 corresponds to the unlabeled metabolite, M+1 indicates one ^13^C atom, M+2 indicates two ^13^C, and so on. For [2-¹³C_1_]ethanol, labeling patterns indicate incorporation of multiple ethanol carbon backbones. The shaded (red) carbons in the chemical diagram, using n-16:0 as an example, indicate potential incorporation sites. For the [1-¹³C_1_]butyrate condition, a dominant M+1 isotopologue indicates intact incorporation of a single butyrate carbon backbone into straight-chain fatty acids, as indicated in the chemical diagram.

When cells were grown in the presence of ^2^H₉-butanol, we observed substantial deuterium incorporation into straight-chain fatty acids with even-numbered carbon lengths, including n-14:0, n-16:0, and n-18:0 (Fig. 2A; Table S2). The presence of a dominant M+7 isotopologue (i.e., a fatty acid molecule containing seven deuterons) indicates intact incorporation of a single *n*-butanol carbon backbone into the fatty acid structure. The predominance of the M+7 species, rather than heavier isotopologues (e.g., M+14), further supports the incorporation of a single, rather than multiple, *n*-butanol-derived units (Fig. 2A; Table 2).

**Fig 3:**
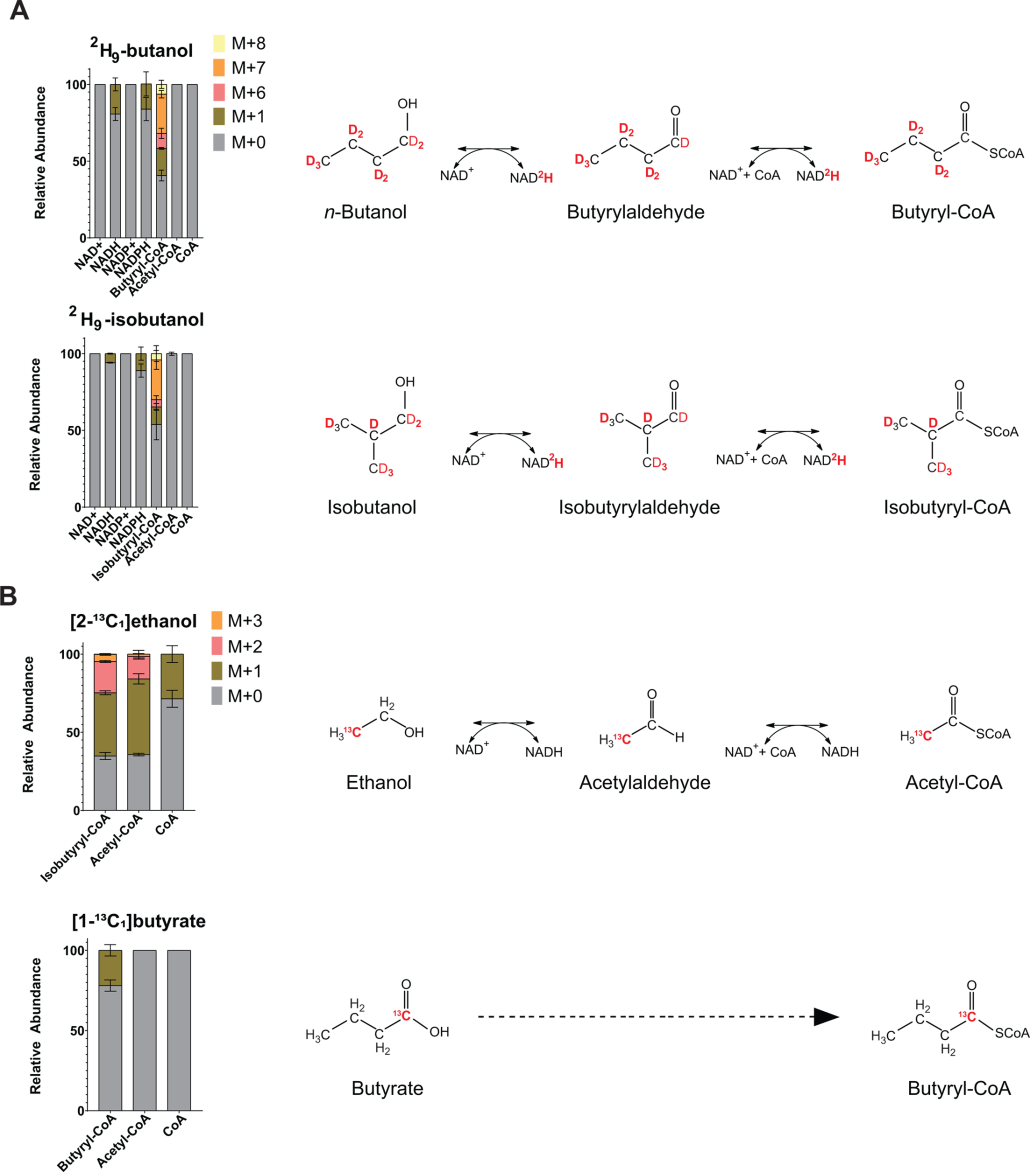
Incorporation of exogenous alcohols and organic acids into fatty acid biosynthetic precursors. Wild-type *C. thermocellum* was grown in minimal media containing cellobiose as the sole carbon source and supplemented with ²H₉-butanol or ²H₉-isobutanol, [1-¹³C_1_]butyrate, or [2-¹³C_1_]ethanol. Bars represent the relative abundance of each isotopologue for the indicated metabolite under each exposure condition. Values are averages of three biological replicates, and error bars indicate standard deviation. Isotopologue distributions for all lipid species measured are shown in Table S14. (**A**) For ²H₉-butanol and ²H₉-isobutanol, M+0 denotes the unlabeled metabolite, M+1 indicates the incorporation of one deuteron, M+2 indicates the incorporation of two deuterons, and so on. Because isobutyryl-CoA and butyryl-CoA share the same LC retention time and molecular mass, their combined mass isotopologue distributions are shown as iso/butyryl-CoA. A dominant M+7 isotopologue in iso/butyryl-CoA indicates assimilation of butanol or isobutanol into butyryl-CoA or isobutyryl-CoA, respectively. The presence of M+1 in NADH, together with the absence of labeling in NAD^+^, suggests transfer of a deuteron from ²H₉-butanol or ²H₉-isobutanol to the active hydride of NADH during oxidation of these alcohols to their corresponding aldehydes and acyl-CoAs. (**B**) For [1-¹³C_1_]butyrate and [2-¹³C_1_]ethanol, M+0 denotes the unlabeled metabolite, M+1 indicates one ¹³C atom, M+2 indicates two ¹³C atoms, and so on. Under the [2-¹³C_1_]ethanol condition, the presence of an M+1 isotopologue in iso/butyryl-CoA indicates assimilation of ethanol into isobutyryl-CoA.

Interestingly, we also observed deuterium labeling in branched-chain fatty acids such as iso-14:0, iso-16:0, iso-17:0, and iso-18:0 in the presence of ^2^H₉-butanol. However, in contrast to the straight-chain species, the labeling pattern in these branched-chain fatty acids was dominated by M+1 species (i.e., containing a single deuteron). This suggests that deuterium incorporation did not result from assimilation of the *n*-butanol carbon backbone, but rather from exchangeable, non-carbon-bound deuterons—likely introduced via deuterated cofactors such as NADPH—during reductive steps of fatty acid biosynthesis. Supporting this explanation, we found that both NADH and NADPH, but not NAD^+^ or NADP^+^, incorporated a single deuteron during ²H₉-butanol exposure, consistent with labeling at the hydride-transfer site (Fig. 3A). Therefore, the presence of M+8 isotopologues in straight-chain fatty acids, in addition to M+7 species, further supports labeling via both the *n*-butanol backbone and deuterons from redox cofactors (Fig. 2A; Table S2).

In contrast to ^2^H₉-butanol, exposure to ^2^H₉-isobutanol resulted in substantial incorporation of deuterium into branched-chain fatty acids, including iso-14:0, iso-16:0, iso-17:0, and iso-18:0. As with *n*-butanol, the presence of a dominant M+7 isotopologue indicates intact incorporation of a single isobutanol carbon backbone into these fatty acids (Fig. 2A; Table S2). In parallel, M+1 labeling observed in straight-chain fatty acids (e.g., n-14:0, n-16:0, and n-18:0) indicates incorporation of non-carbon-bound deuterons from redox cofactors, as NADH and NADPH also incorporated a single deuteron during ^2^H₉-isobutanol exposure (Fig. 3A). Thus, the presence of M+8 isotopologues in branched-chain fatty acids, in addition to M+7 species, supports labeling via both the isobutanol backbone and exchangeable deuterons from redox cofactors (Fig. 2A).

Exposure to [1-¹³C_1_]butyrate resulted in pronounced incorporation of ^13^C into straight-chain fatty acids, with negligible labeling in branched-chain fatty acids. The appearance of a single M+1 isotopologue (containing one ^13^C atom) was consistent with the incorporation of a single butyrate-derived unit into the fatty acid backbone (Fig. 2B; Table S2).

Finally, [2-¹³C_1_]ethanol exposure resulted in extensive ^13^C labeling of both straight-chain and branched-chain fatty acids. Unlike the other tested substrates, ethanol yielded a broad distribution of isotopologues (ranging from M+1 to M+7), indicating that multiple ethanol-derived units can be incorporated during fatty acid biosynthesis (Fig. 2B; Table S2).

Collectively, these results demonstrate that *C. thermocellum* can directly incorporate the carbon backbones of exogenous alcohols and organic acids into fatty acids, providing a mechanistic basis for the opposing shifts in straight-chain versus iso-branched fatty acid composition observed under *n*-butanol, butyrate, and isobutanol exposure. In addition, incorporation of non-carbon-bound deuterons—derived from deuterated cofactors such as NADPH— was observed across both straight-chain and branched-chain fatty acids.

### Exogenous alcohols and acids are assimilated into membrane lipids

To further confirm the incorporation of exogenous alcohol- and acid-derived carbon backbones into membrane lipids, we performed LC-MS/MS analysis of *C. thermocellum* lipid extracts (see Materials and Methods). The major membrane lipid classes identified were monogalactosyldiacylglycerol (MGDG), phosphatidylglycerol (PG), diacylglycerol (DG), digalactosyldiacylglycerol (DGDG), and PG plasmalogens (Table S4). Diacyl lipids (MGDG, PG, DG, and DGDG) contain two ester-linked fatty acyl chains, whereas PG plasmalogens contain a vinyl-ether-linked plasmenyl chain at the sn-1 position and an ester-linked fatty acyl chain at the sn-2 position. Each lipid species was observed with either two branched-chain acyl groups, two straight-chain acyl groups, or one branched and one straight-chain acyl group.

**Fig 4:**
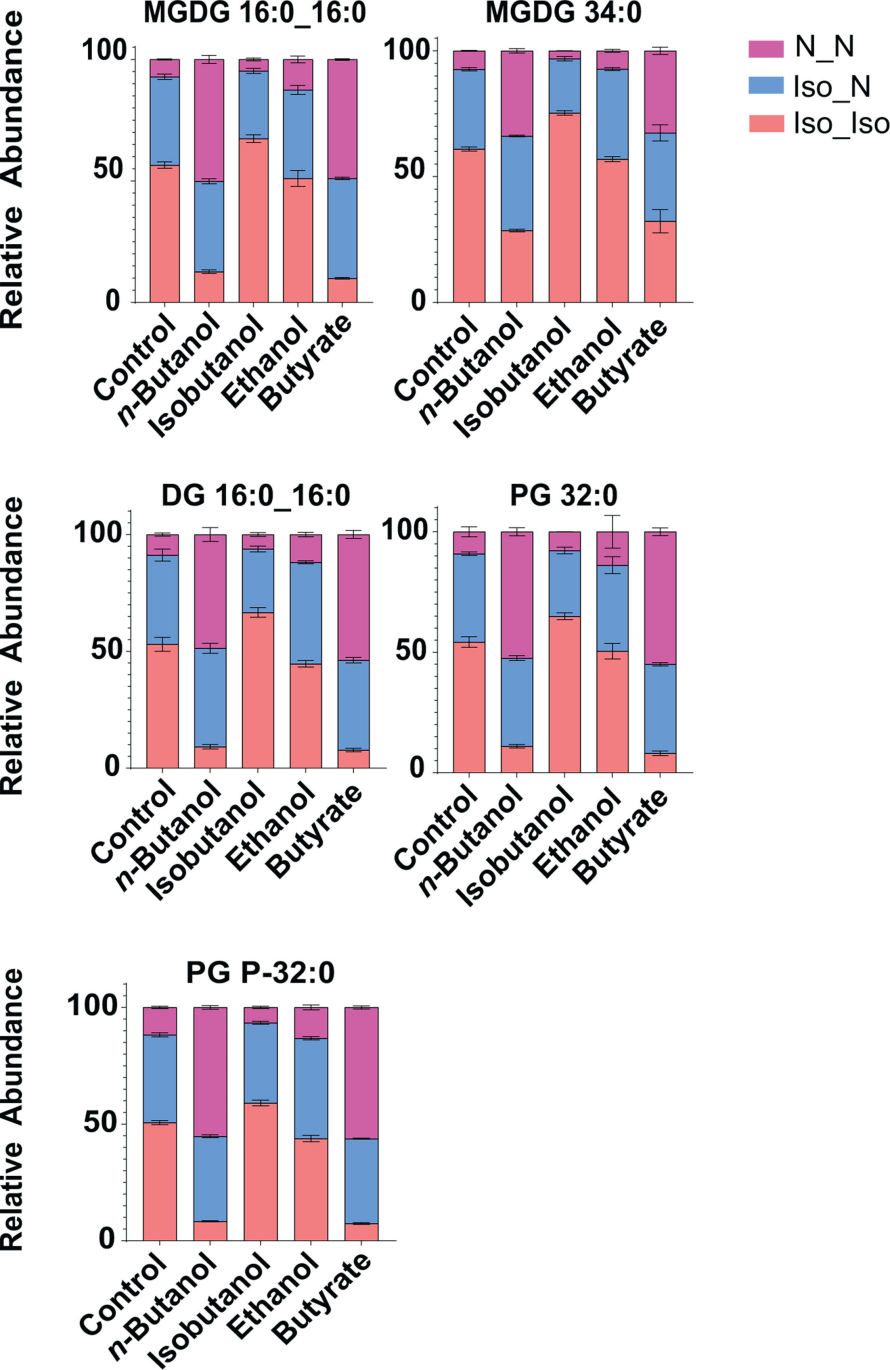
Exposure to alcohols and organic acids alters acyl chain composition of membrane lipids. Wild-type *C. thermocellum* (DSM1313) was grown in minimal media containing cellobiose as the sole carbon source and supplemented with n-butanol, isobutanol, ethanol, or butyrate. Bar graphs show the relative acyl chain composition of representative lipid species under the indicated exposure conditions. Data represent the average of three biological replicates, with error bars indicating standard deviation. N_N denotes two straight-chain acyl groups, Iso_Iso denotes two branched-chain acyl groups, and Iso_N denotes one branched-chain and one straight-chain acyl group. Acyl chain composition of all lipid species measured is shown in Table S4. *n*-Butanol and butyrate exposure markedly increased the relative proportion of lipid species containing two straight-chain acyl groups. In contrast, isobutanol exposure increased the proportion of lipids containing branched-chain acyl groups. Abbreviations: Diacylglycerols (DG), Monogalactosyldiacylglycerol (MGDG), Phosphatidylglycerol (PG), Plasmenyl phosphatidylglycerol (PG-P).

As we anticipated, shifts in fatty acid composition observed under solvent and acid exposure were reflected in the acyl chain composition of membrane lipids (Fig. 4; Table S4). Specifically, *n*-butanol and butyrate exposure markedly increased the relative proportion of lipid species containing straight-chain acyl groups (i.e., n-16:0 and n-18:0) across all lipid classes examined. Conversely, isobutanol exposure led to a modest increase in the proportion of lipids containing branched-chain acyl groups (i.e., iso-16:0 and iso-18:0). Ethanol exposure had minimal impact on lipid acyl chain composition, consistent with the relatively modest changes observed in total fatty acid pools (Fig. 4; Table S4).

**Fig 5:**
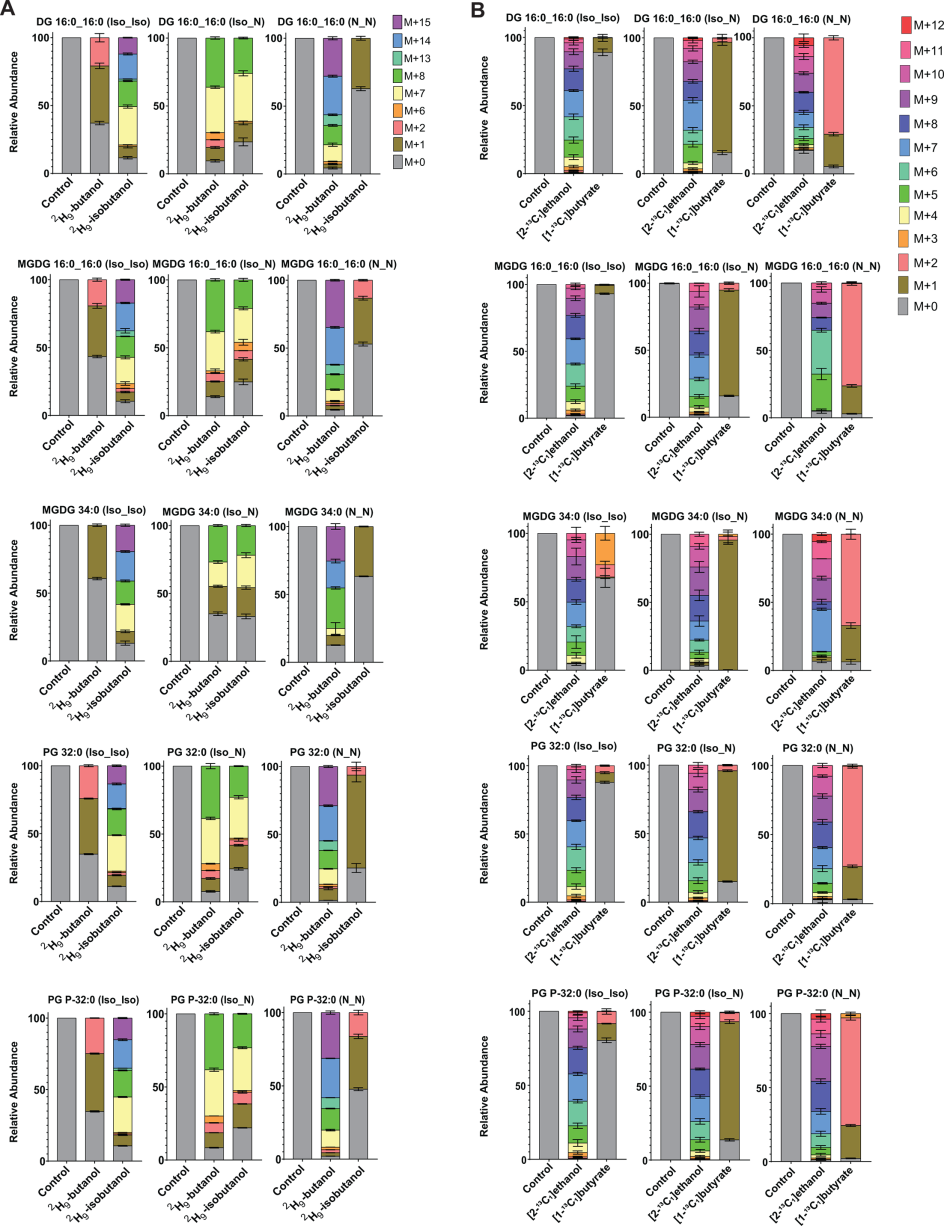
Isotopic labeling shows incorporation of exogenous alcohols and organic acids into membrane lipids. Wild-type *C. thermocellum* was grown in minimal media containing cellobiose as the sole carbon source and supplemented with ²H₉-butanol or ²H₉-isobutanol, [1-¹³C_1_]butyrate, or [2-¹³C_1_]ethanol. Bars show the relative abundance of each isotopologue for individual lipid species under the indicated exposure conditions. Isotopologue distributions for all lipid species measured are shown in Table S3. Values represent the average of three biological replicates, with error bars indicating standard deviation. N_N denotes two straight-chain acyl groups, Iso_Iso denotes two branched-chain acyl groups, and Iso_N denotes one branched-chain and one straight-chain acyl group. (**A**) M+0 corresponds to the unlabeled metabolite, M+1 indicates the incorporation of one deuteron, M+2 indicates the incorporation of two deuterons, and so on. The presence of M+7 indicates incorporation of a single butanol- or isobutanol-derived carbon backbone into one fatty acyl chain, while M+14 indicates incorporation into both acyl chains. M+1 and M+2 species reflect incorporation of exchangeable deuterons from deuterated NADPH, while heavier species (M+8, M+15, and others) reflect incorporation of both the alcohol carbon backbone and NADPH-derived deuterons. (**B**) M+ 0 corresponds to the unlabeled metabolite; M+1, M+2, and higher values indicate incorporation of one, two, or more ^13^C atoms. For [1-¹³C_1_]butyrate, M+1 indicates incorporation of a single butyrate-derived carbon backbone into one fatty acyl chain, while M+2 indicates incorporation into both acyl chains. For [2-¹³C_1_]ethanol, the distribution of isotopologues reflects variable and multiple incorporation of ethanol-derived carbon backbones into fatty acyl chains. Abbreviations: Diacylglycerol (DG), Monogalactosyldiacylglycerol (MGDG), Phosphatidylglycerol (PG), Plasmenyl phosphatidylglycerol (PG-P).

Mirroring the labeling profiles observed in fatty acids, cultures grown with ²H₉-butanol exhibited substantial deuterium incorporation (M+7 and heavier isotopologues) into lipids containing straight-chain acyl groups but not in those containing exclusively branched-chain acyl groups (Fig. 5A; Table S3). Similarly, [1-¹³C_1_]butyrate led to pronounced ¹³C incorporation in lipids containing straight-chain acyl groups, with negligible labeling in those containing only branched-chain acyl groups. In contrast, ²H₉-isobutanol resulted in large deuterium incorporation into lipids containing branched-chain acyl groups but not those containing solely straight-chain acyl groups. As expected, [2-¹³C_1_]ethanol resulted in widespread ¹³C incorporation across all lipid species, independent of acyl chain branching (Fig. 5B; Table S3).

Together, these data demonstrate that *C. thermocellum* can directly assimilate the carbon backbones of exogenous alcohols and organic acids into fatty acids, which are then incorporated into diverse membrane lipid classes.

### AdhE is required for the incorporation of exogenous alcohols into membrane lipids

In *C. thermocellum*, AdhE is a bifunctional aldehyde/alcohol dehydrogenase that catalyzes the NADH-dependent reduction of acetyl-CoA to ethanol via the intermediate acetaldehyde (23–26). We hypothesize that exogenous alcohols such as *n*-butanol and isobutanol can be incorporated into fatty acids through the oxidative activity of AdhE. In this proposed pathway, AdhE oxidizes these alcohols to their corresponding aldehydes, which are subsequently converted to the acyl-CoA intermediates butyryl-CoA and isobutyryl-CoA. These intermediates would then serve as substrates for fatty acid biosynthesis.

**Fig 6:**
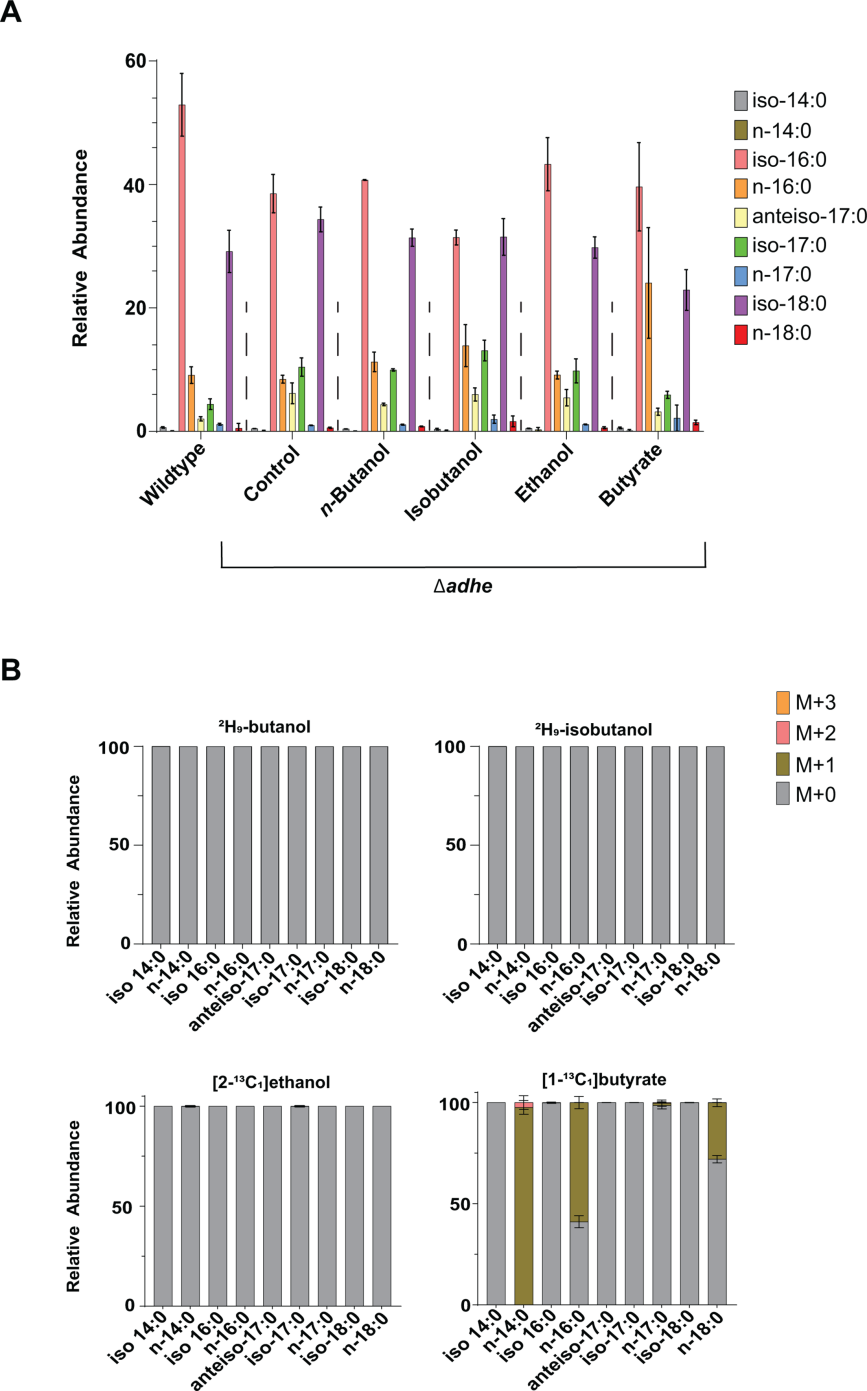
AdhE is required for the incorporation of exogenous alcohols into fatty acids. (**A**) *C. thermocellum* Δ*adhE* strain, lacking the bifunctional aldehyde/alcohol dehydrogenase, was grown in minimal medium supplemented with n-butanol, isobutanol, ethanol, or butyrate, as well as in control medium without added alcohols or acids. (**A**) Bars show the relative abundance of individual fatty acids within the total fatty acid pool under the indicated exposure conditions. Values represent the average of three (n-butanol, isobutanol, and butyrate) or two (ethanol) biological replicates, with error bars indicating standard deviation. In contrast to the substantial remodeling observed in the wild-type strain (Fig. 1), supplementation with n-butanol or isobutanol produced only minor effects on fatty acid composition in the Δ*adhE* strain. However, butyrate still significantly increased the abundance of n-16:0 at the expense of branched-chain fatty acids. Statistical comparisons (two-tailed t-test) of the Δ*adhE* strain under supplemented versus unsupplemented conditions, as well as relative to the wild-type strain, are reported in Table S5. (**B**) The bar graphs show the relative abundance of isotopologues for individual fatty acids under the indicated exposure conditions. Values represent the average of three (n-butanol, isobutanol, and butyrate) or two (ethanol) biological replicates, with error bars indicating standard deviation. For ²H₉-butanol and ²H₉-isobutanol, M+0 corresponds to the unlabeled metabolite, M+1 indicates the incorporation of one deuteron, M+2 indicates the incorporation of two deuterons, and so on. For [1-¹³C_1_]butyrate and [2-¹³C_1_]ethanol, M+0 denotes the unlabeled metabolite, M+1 indicates one ¹³C atom, M+2 indicates two ¹³C atoms, and so on. In contrast to the wild-type strain (Fig. 2), no incorporation of deuterium from ²H₉-butanol or ²H₉-isobutanol into fatty acids was detected in the Δ*adhE* strain. Unlike alcohols, [1-¹³C_1_]butyrate was still readily incorporated into straight-chain fatty acids and membrane lipids (Table S6) in the Δ*adhE* strain, as evidenced by prevalent M+1 labeling.

To test this hypothesis, we exposed a *C. thermocellum* Δ*adhE* mutant strain to ^2^H₉-butanol, ^2^H₉-isobutanol, [2-^13^C_1_]ethanol, and [1-^13^C_1_]butyrate. The fatty acid profile of the Δ*adhE* strain was largely similar to that of the wild-type, although it exhibited a modest decrease in iso-16:0 fatty acids and an increase in iso-17:0 and anteiso-17:0 species (Fig. 6A; Table S5).

Unlike the substantial changes observed in the wild-type strain, supplementation with *n*-butanol or isobutanol did not substantially alter the fatty acid composition in the Δ*adhE* strain. However, the addition of butyrate still led to a pronounced increase in straight-chain fatty acids, particularly n-16:0, along with a corresponding rise in membrane lipids containing straight-chain acyl groups (Fig. 6A; Fig. S1).

Isotope tracer experiments revealed that deletion of *adhE* eliminated incorporation of carbon backbones from *n*-butanol, isobutanol, and ethanol into fatty acids and membrane lipids (Fig. 6B; Fig. S2 and Table S6). In contrast, labeled butyrate was still readily incorporated into straight-chain fatty acids and their corresponding membrane lipids in the Δ*adhE* strain (Fig. 6B; Fig. S2).

These findings support the hypothesis that AdhE facilitates the assimilation of exogenous alcohols into fatty acids by oxidizing them first to aldehydes and then to CoA-linked acyl intermediates, which are subsequently incorporated into fatty acid metabolism. Further support comes from the isotopic labeling patterns of metabolic intermediates: in wild-type cells, exposure to ²H₉-butanol and ²H₉-isobutanol led to the formation of M+7 isotopologues of butyryl-CoA and isobutyryl-CoA, consistent with incorporation of the deuterated carbon backbone of *n*-butanol and isobutanol into these intermediates (Fig. 3A).

### Phosphate acetyltransferase plays a major role in butyrate assimilation into fatty acids

While AdhE is essential for alcohol assimilation, the Δ*adhE* strain retained the ability to incorporate butyrate into central metabolism and membrane lipids, indicating the presence of an alternative route for organic acid uptake. One plausible mechanism is the reversal of the acetate fermentation pathway. In *C. thermocellum*, acetate is produced via a two-step process: phosphate acetyltransferase (Pta) converts acetyl-CoA to acetyl-phosphate, which is subsequently converted to acetate by acetate kinase (Ack) (27). If these enzymes exhibit substrate promiscuity, exogenous butyrate could be converted to butyryl-phosphate by Ack and subsequently to butyryl-CoA by Pta. Consistent with this possibility, cells grown with [1-¹³C] butyrate displayed a predominant M+1 isotopologue of butyryl-CoA, indicating incorporation of the labeled carboxyl carbon (Fig. 3B).

**Fig 7:**
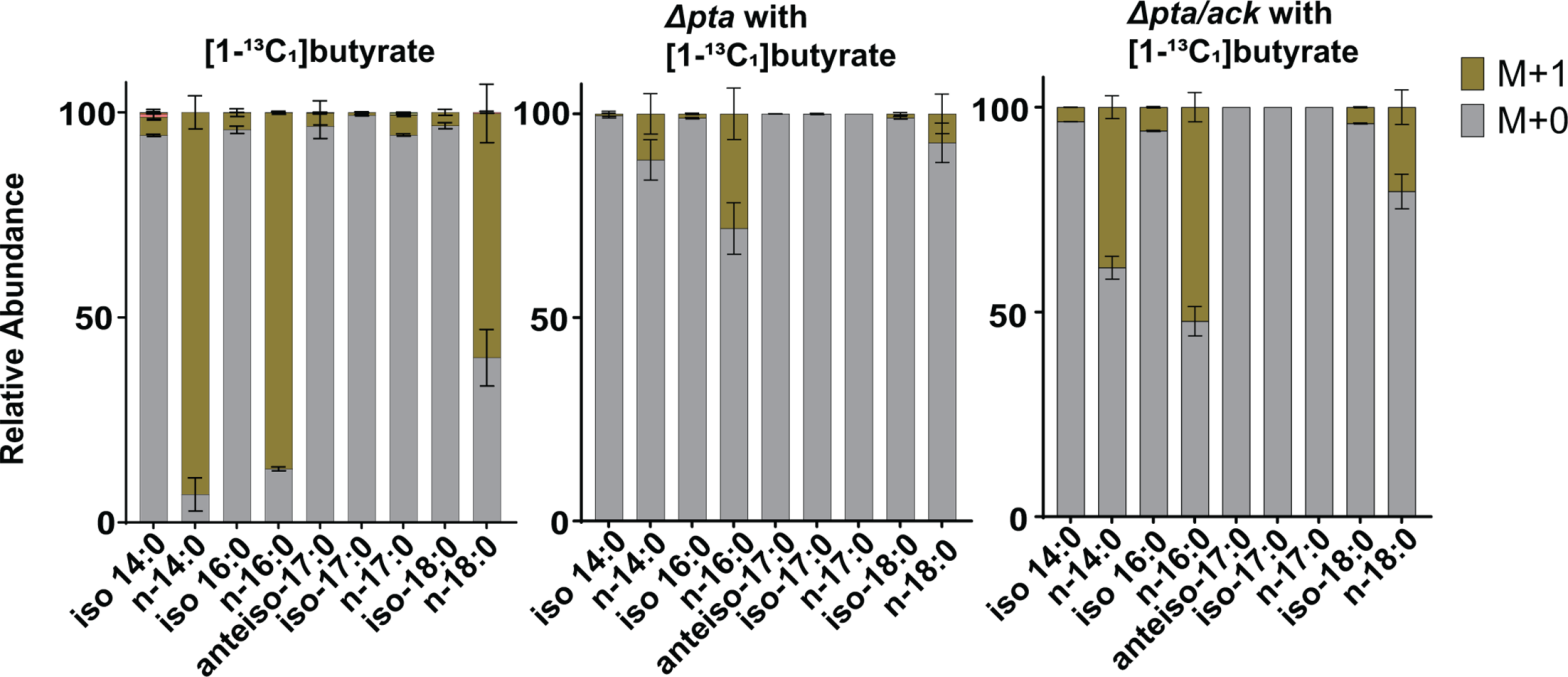
Butyrate uptake is substantially reduced in the absence of Pta or Ack. Isotopologue distribution of fatty acids in *C. thermocellum* strains lacking Pta (Δ*pta*) or both Pta and Ack (Δ*pta/ack*) when supplemented with [1-¹³C_1_]butyrate. For comparison, the wild-type strain is also shown. Bars show the relative abundance of each isotopologue for individual fatty acids. Values represent the average of three biological replicates, with error bars indicating standard deviation. M+0 corresponds to the unlabeled metabolite, M+1 indicates the incorporation of one ^13^C atom. Isotopologue distributions of measured fatty acids are provided in Table S18.

To test this hypothesis directly, we exposed *C. thermocellum* strains lacking Pta (Δ*pta*) or both Pta and Ack (Δ*pta/ack*) to exogenous [1-¹³C_1_]butyrate (Fig. 7; Tables S17 and 18). Compared to wild-type, deletion of Pta drastically reduced the extent of incorporation of ^13^C to n-14:0 (85%–15%), n-16:0 (75%–23%), and n-18:0 (60%–10%) fatty acids, suggesting that Pta participates in a major route of butyrate assimilation into fatty acids. The Δ*pta/ack* double mutant also showed substantially reduced incorporation into these fatty acids; however, the reduction was consistently smaller than that observed in the Δ*pta* strain. These results indicate that, although Pta plays a dominant role in butyrate assimilation into fatty acids, additional Pta- and Ack-independent route(s) also mediate conversion of butyrate into fatty acid precursors. This interpretation is consistent with prior observations that both Δ*pta* and Δ*pta*/*ack* strains retain measurable acetate production, with the double mutant producing slightly more acetate than the single mutant (28, 29).

### Pyruvate ferredoxin oxidoreductase 4 (Pfor4) is essential for branched fatty acid synthesis

Pyruvate:ferredoxin oxidoreductase (Pfor) is a multi-subunit enzyme complex that catalyzes the oxidative decarboxylation of pyruvate to acetyl-CoA, coupling this reaction to the reduction of ferredoxin. The genome of *C. thermocellum* encodes five annotated Pfor complexes (*pfor1* to *pfor5*). Previous studies have shown that *pfor1* (Clo1313_0020-0023) and *pfor4* (Clo1313_1353-1356) are cumulatively responsible for approximately 80% of Pfor activity, indicating a major role in ethanol fermentation (30, 31). Consistent with this, recent proteomic analyses indicate that Pfor1 and Pfor4 are the most highly expressed Pfor complexes, with Pfor3 also exhibiting high expression levels (30).

**Fig 8:**
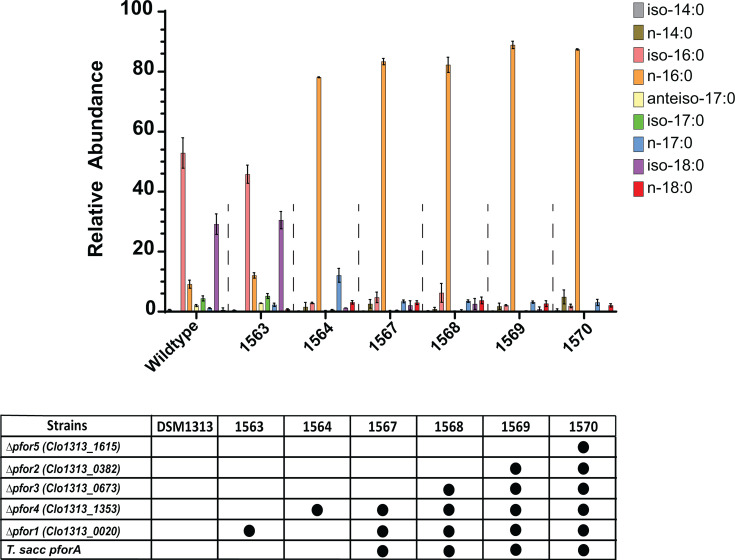
Pyruvate:ferredoxin oxidoreductase 4 (Pfor4) is required for branched-chain fatty acid synthesis in *C. thermocellum*. Fatty acid profiles of *C. thermocellum* strains carrying sequential deletions of *pfor* genes. Bars represent the relative abundance of individual fatty acids within the total fatty acid pool under the indicated conditions. Values represent the average of three (LL1563 and LL1570) or two (LL1564, LL1567, LL1568, and LL1569) biological replicates, with error bars showing standard deviation. Deletion of *pfor1* (Clo1313_0020) had little effect on membrane fatty acid composition, whereas deletion of *pfor4* (Clo1313_1353) caused major shifts: iso-16:0 and iso-18:0 were nearly absent, iso-17:0 and anteiso-17:0 were eliminated, and straight-chain fatty acids, especially n-16:0, increased markedly. Further deletion of *pfor2*, *pfor3*, or *pfor5* in the *Δpfor1 Δpfor4* background did not significantly alter fatty acid composition beyond the effects of *pfor4* deletion. The expression of *T. saccharolyticum pforA* in strains 1567–1570 preserves the pyruvate-to–acetyl-CoA flux that would otherwise be nearly eliminated by deletion of both *pfor1* and *pfor4*. Statistical significance of changes in fatty acid abundance (two-tailed t-test) in *pfor* deletion strains relative to wild-type is reported in Table S9. Strains with more than two *pfor* deletions carry a heterologous copy of *pforA* from *T. saccharolyticum*. Abbreviations: Δ*pfor1* (*Clo1313_0020–0023*); Δ*pfor2* (*Clo1313_0382–0385*); Δ*pfor3* (*Clo1313_0673*); Δ*pfor4* (*Clo1313_1353–1356*); and Δ*pfor5* (*Clo1313_1615–1616*).

Because acetyl-CoA is a key precursor for fatty acid biosynthesis, we investigated the role of Pfor complexes in modulating membrane lipid composition. To test this, we analyzed the membrane fatty acid profiles of a series of *C. thermocellum* strains in which the five *pfor* gene clusters were sequentially deleted (Fig. 8). Deletion of *pfor1* (i.e., Clo1313_0020) did not significantly alter the membrane fatty acid composition relative to wild-type. In contrast, deletion of *pfor4* (i.e., Clo1313_1353) caused dramatic changes: the branched-chain fatty acids iso-16:0 and iso-18:0, which predominate in wild-type membranes, were nearly absent; iso-17:0 and anteiso-17:0 fatty acids disappeared entirely; while straight-chain fatty acids, especially n-16:0, increased markedly. The combined deletion of *pfor1* and *pfor4* resulted in a fatty acid profile similar to the *pfor4* single deletion strain, dominated by n-16:0 and showing a near absence of branched-chain fatty acids. Further deletion of *pfor3* (i.e., Clo1313_0673), *pfor2* (Clo1313_0382), and *pfor5* (Clo1313_1615) on top of the *pfor1*/*pfor4* deletions did not produce additional significant changes in fatty acid composition. These findings show that Pfor4 plays a critical and non-redundant role in the synthesis of both even-chain (iso-16:0 and iso-18:0) and odd-chain branched (iso-17:0 and anteiso-17:0) fatty acids in *C. thermocellum*.

In bacteria, the canonical biosynthesis of branched-chain fatty acids involves the diversion of branched-chain α-keto acid intermediates from amino acid biosynthesis. The compounds 2-oxoisovalerate, 2-oxo-3-methylvalerate, and 2-oxocaproate can either be transaminated to form the branched-chain amino acids valine, isoleucine, and leucine, respectively, or be oxidatively decarboxylated to form the corresponding acyl-CoA intermediates (32, 33). This decarboxylation step is catalyzed by the branched-chain α-keto acid dehydrogenase (Bkd) complex, which converts 2-oxoisovalerate to isobutyryl-CoA, 2-oxo-3-methylvalerate to 2-methylbutyryl-CoA, and 2-oxocaproate to isovaleryl-CoA (32, 33). These acyl-CoA intermediates then serve as primers for branched-chain fatty acid biosynthesis: isobutyryl-CoA gives rise to even-chain iso-branched fatty acids, 2-methylbutyryl-CoA to odd-chain anteiso-branched fatty acids, and isovaleryl-CoA to odd-chain iso-branched fatty acids. Our data suggest that in *C. thermocellum*, Pfor4 functionally substitutes for the Bkd complex in branched-chain fatty acid biosynthesis, explaining the near depletion or absence of both even- and odd-chain branched fatty acids observed upon its deletion.

### Exogenous isobutanol restores branched-chain fatty acid synthesis in the absence of Pfor4

Our results indicate that *C. thermocellum* possesses two potential pathways for the synthesis of branched-chain fatty acids: (i) a Pfor4-dependent route that decarboxylates branched-chain α-keto acids derived from amino acid synthesis and (ii) an alternative route in which exogenous branched-chain alcohols are oxidized to acyl-CoA intermediates via oxidative AdhE activity, enabling their incorporation into fatty acids.

**Fig 9:**
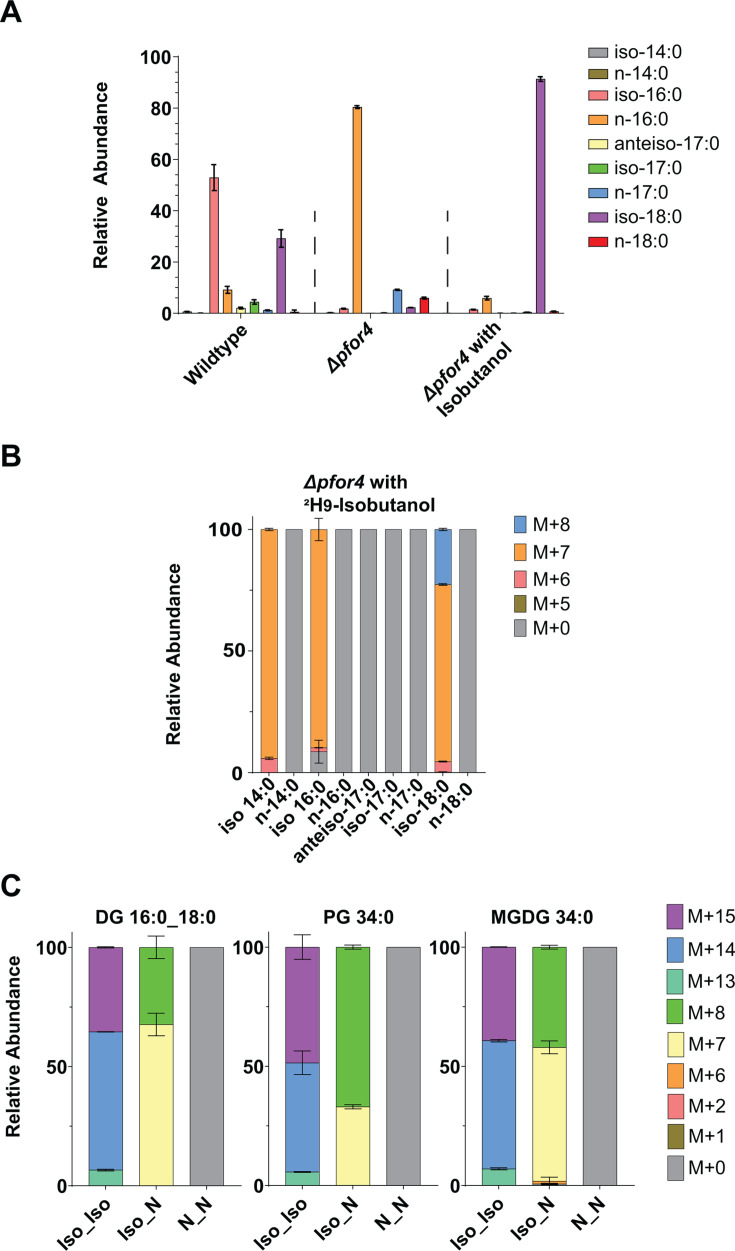
Isobutanol exposure restores branched-chain fatty acid synthesis in a Δ*pfor4* strain. (**A**) Fatty acid profiles of wild-type *C. thermocellum*, the Δ*pfor4* strain, and the Δ*pfor4* strain supplemented with isobutanol. Bars represent the relative abundance of individual fatty acids within the total fatty acid pool. Data represent average values from three biological replicates, with error bars indicating standard deviation. Statistical significance of changes in fatty acid abundance (two-tailed t-test) is reported in Table S10. (**B**) Isotopologue distribution of fatty acids in the Δ*pfor4* strain supplemented with ²H₉-isobutanol. Bars show the relative abundance of each isotopologue for individual fatty acids. Values represent the average of three biological replicates, with error bars indicating standard deviation. M+0 corresponds to the unlabeled metabolite, M+1 indicates the incorporation of one deuteron, M+2 indicates the incorporation of two deuterons, and so on. Labeling patterns demonstrate incorporation of a single isobutanol carbon backbone into iso-fatty acids with an even number of carbons (iso-14:0, iso-16:0, and iso-18:0). Isotopologue distributions of measured fatty acids can be found in Table S11. (**C**) Isotopologue distribution of membrane lipids in the Δ*pfor4* strain supplemented with ²H₉-isobutanol. Bars show the relative abundance of each isotopologue for representative lipid species. Values represent the average of three biological replicates, with error bars indicating standard deviation. N_N denotes two straight-chain acyl groups, Iso_Iso denotes two branched-chain acyl groups, and Iso_N denotes one branched-chain and one straight-chain acyl group. M+0 corresponds to the unlabeled metabolite, M+1 indicates the incorporation of one deuteron, M+2 indicates the incorporation of two deuterons, and so on. The presence of M+7 indicates incorporation of a single isobutanol-derived carbon backbone into one fatty acyl chain, while M+14 indicates incorporation into both acyl chains. Heavier species (M+8, M+15) represent incorporation of both the isobutanol backbone and NADPH-derived deuterons. Isotopologue distributions of measured acyl-chain lipids can be found in Table S12. Abbreviations: Diacylglycerol (DG), Monogalactosyldiacylglycerol (MGDG), Phosphatidylglycerol (PG).

If these pathways provide redundant routes to branched-chain acyl-CoAs, we hypothesized that supplementing the growth medium with exogenous branched-chain alcohols should restore branched-chain fatty acid synthesis in ∆*pfor4* strains. To test this, we grew the ∆*pfor4* strain in medium supplemented with 3 g/L isobutanol and analyzed fatty acid and lipid composition using LC-MS/MS (see Materials and Methods). In the absence of isobutanol, the ∆*pfor4* strain exhibited severely depleted levels of branched-chain fatty acids. However, isobutanol addition dramatically restored iso-fatty acid production, with iso-18:0 comprising over 90% of the total fatty acid pool (Fig. 9A; Table S10).

This shift in fatty acid composition was mirrored in the acyl chain profile of membrane lipids (Fig. S3). In the Δ*pfor4* strain, membrane lipids contained almost exclusively straight-chain acyl groups (>90% in most cases). However, upon exposure to isobutanol, this pattern reversed, and branched-chain acyl groups became predominant, accounting for 80% to 90% of the total lipid acyl chains (Fig. 9C; Fig. S3 and Table S12).

Labeling experiments with ²H₉-isobutanol confirmed that the intact carbon backbone of isobutanol was incorporated into the branched-chain fatty acids iso-14:0, iso-16:0, and iso-18:0, as evidenced by a predominant M+7 isotopologue. In contrast, no labeling was detected in straight-chain or odd-chain fatty acids (Fig. 9B; Table S11). Similarly, lipids containing branched-chain acyl groups exhibited substantial deuterium enrichment, showing both M+7 and M+14 isotopologues, whereas lipids composed exclusively of straight-chain acyl groups did not.

Together, these results show that exogenous isobutanol can bypass the requirement for Pfor4 by serving as a precursor for branched-chain fatty acid synthesis via AdhE-mediated conversion to isobutyryl-CoA.

## DISCUSSION

In this study, we show that the membrane lipid composition of *C. thermocellum* changes markedly in response to exogenous alcohols and organic acids. Exposure to linear alcohols, such as *n-*butanol, or organic acids, such as butyrate, shifts the acyl chain profile of membrane lipids toward a higher proportion of n-fatty acids at the expense of branched-chain species, whereas exposure to branched alcohols such as isobutanol has the opposite effect.

**Fig 10:**
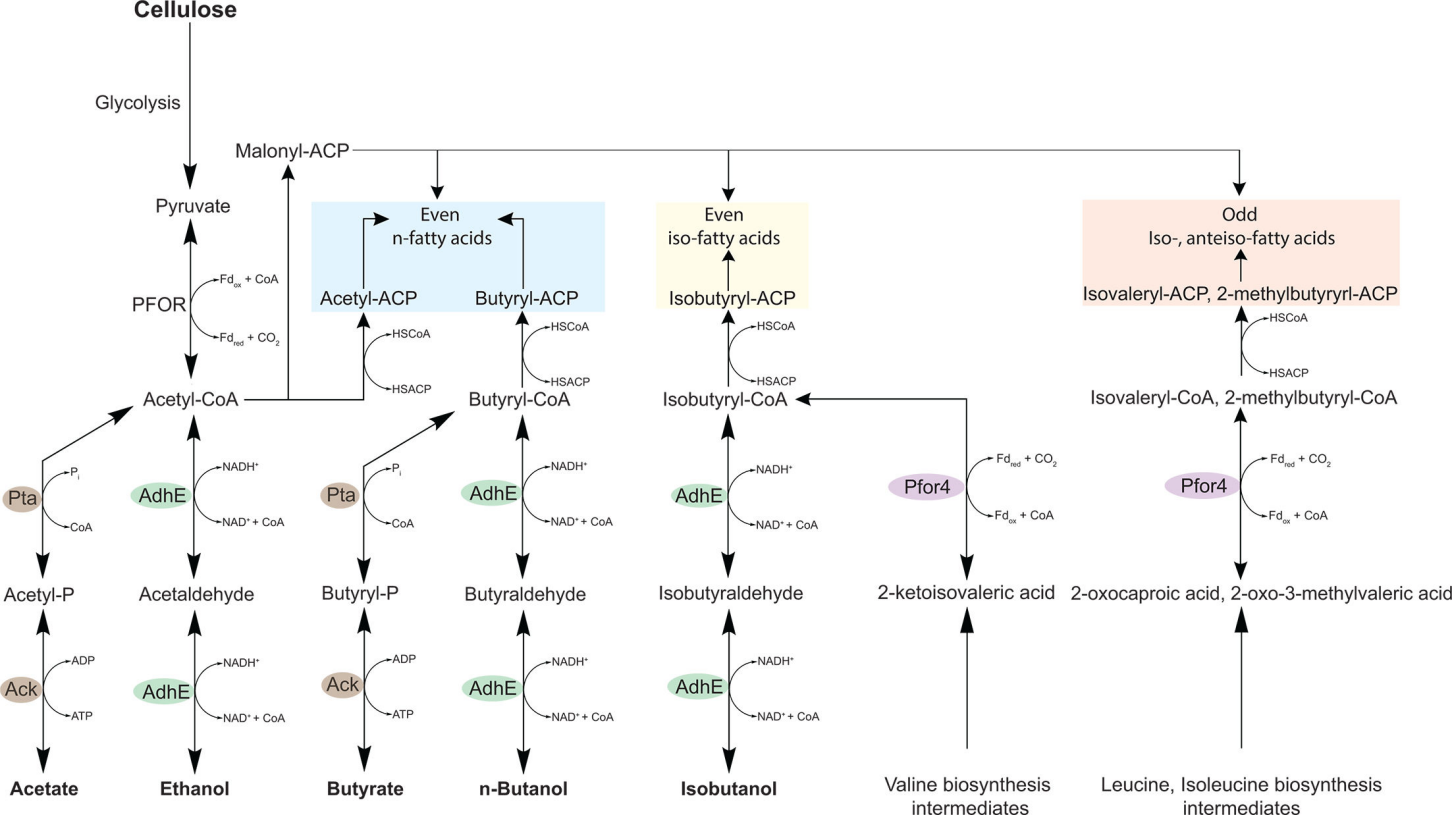
Proposed roles of AdhE and Pfor4 in fatty acid biosynthesis in *C. thermocellum*. Metabolic diagram illustrating the pathways for straight-chain and branched-chain fatty acid biosynthesis in *C. thermocellum*, including newly proposed routes. The bifunctional aldehyde/alcohol dehydrogenase AdhE assimilates exogenous alcohols through its oxidative activity: oxidizing alcohols to the corresponding aldehydes and subsequently converting them into acyl-CoA intermediates. These intermediates can then be incorporated into straight- and branched-chain fatty acid synthesis via their conversion to acyl-ACPs. Pfor4 decarboxylates branched-chain α-keto acids derived from amino acid metabolism to generate acyl-CoA intermediates for branched-chain fatty acid synthesis, a role typically performed by the branched-chain α-keto acid dehydrogenase (Bkd) complex in many bacteria. Abbreviations: Pyruvate:ferredoxin oxidoreductase (Pfor), Alcohol dehydrogenase E (AdhE), Acetate kinase (Ack), Phosphate acetyltransferase (Pta), Acyl-carrier protein (ACP), Coenzyme A (CoA), Nicotinamide adenine dinucleotide (NAD^+^/NADH), Ferredoxin (Fd_ox_, Fd_red_), Inorganic phosphate (Pi).

Isotope tracer experiments revealed that *C. thermocellum* directly incorporates the carbon backbone of exogenous alcohols and organic acids into fatty acids, providing a mechanistic explanation for the contrasting shifts in straight-chain versus iso-branched fatty acid composition observed under *n*-butanol, butyrate, and isobutanol exposure (Fig. 10).

We further show that the bifunctional aldehyde/alcohol dehydrogenase AdhE is essential for the assimilation of exogenous alcohols through its oxidative activity: oxidation of alcohols to the corresponding aldehydes, followed by conversion to acyl-CoA intermediates. We also show that Pta, in the acetate fermentation pathway, plays a predominant role in butyrate assimilation into fatty acid precursors. These conclusions are consistent with previous reports that the ethanol and acetate pathways in *C. thermocellum* are highly reversible (7, 10, 11, 34). Further supporting our proposed mechanism for alcohol assimilation, previous *in vitro* enzyme assays have demonstrated that AdhE can catalyze both the reduction of butyryl-CoA to butyraldehyde and isobutyryl-CoA to isobutyraldehyde (23). Given the high reversibility of the ethanol and acetate fermentation pathways and the broad substrate specificity of the enzymes involved, as suggested by this study, it is likely that *C. thermocellum* can also incorporate into fatty acids other low-molecular weight alcohols and organic acids not examined here, such as propanol, propionate, acetate, and potentially branched and linear five-carbon alcohols and acids.

Isotope tracer data show that the carbon backbones of *n*-butanol, isobutanol, and butyrate are incorporated only once into fatty acids, indicating that their assimilation occurs through conversion from acyl-CoA intermediates into acyl-ACPs (catalyzed by ACP transacylase) and the initial condensation reaction with malonyl-ACP (catalyzed by β-ketoacyl-ACP synthase), rather than during subsequent elongation cycles (Fig. 10). In contrast, ethanol is incorporated multiple times into fatty acids, consistent with its conversion to acetyl-CoA, which serves as the precursor for both acetyl-ACP and malonyl-ACP (Fig. 10).

Our results also indicate that in *C. thermocellum*, the pyruvate:ferredoxin oxidoreductase isozyme Pfor4 (Clo1313_1353-1356) substitutes for the canonical branched-chain α-keto acid dehydrogenase (Bkd) complex in branched-chain fatty acid biosynthesis. Deletion of *pfor4* eliminates both even- and odd-chain branched fatty acids, but supplementation with exogenous isobutanol restores the synthesis of even-chain branched fatty acids. This suggests two possible routes for branched-chain fatty acid production in *C. thermocellum*: (i) a Pfor4-dependent pathway that decarboxylates branched-chain α-keto acids derived from amino acid biosynthesis and (ii) an AdhE-dependent salvage pathway in which exogenous branched-chain alcohols are oxidized to acyl-CoA intermediates, allowing their incorporation into fatty acids.

Bkd genes are absent from the *C. thermocellum* genome, and our bioinformatic searches failed to identify homologs. The canonical Bkd complex comprises three catalytic components—E1 (α-ketoacid decarboxylase), E2 (dihydrolipoyl transacylase), and E3 (dihydrolipoamide dehydrogenase)—that sequentially decarboxylate α-keto acids, transfer the acyl group to CoA, and regenerate the oxidized lipoyl group (33). Pfor enzymes catalyze an analogous TPP-dependent oxidative decarboxylation of α-keto acids (e.g., pyruvate) to yield acyl-CoA, but use ferredoxin rather than NAD^+^/FAD as the electron acceptor (35) (Fig. S4). The *pfor4* locus (Clo1313_1353–1356) encodes proteins with functions analogous to Bkd subunits: a 4Fe–4S ferredoxin-binding domain protein (*Clo1313_1353,* E3-like), a pyruvate flavodoxin/ferredoxin oxidoreductase domain protein (*Clo1313_1354,* E3-like), a TPP-binding domain protein (*Clo1313_1355,* E1-like), and a pyruvate/ketoisovalerate oxidoreductase catalytic domain (*Clo1313_1353,* E2-like). These shared structural and catalytic features support the conclusion that Pfor4 functionally replaces Bkd in *C. thermocellum*. Notably, the *pfor4* locus has also been annotated as a ketoisovalerate ferredoxin-dependent reductase, and previous studies have shown its role in isobutanol synthesis (5, 36). However, its potential contribution to branched-chain fatty acid biosynthesis has not previously been proposed or examined. Future studies involving detailed *in vitro* biochemical characterization of Pfor4 will be important to define its complete substrate range and to clarify the extent to which its activity is directed toward the oxidative decarboxylation of pyruvate to form acetyl-CoA, if at all, versus branched-chain fatty acid synthesis.

The membrane fatty acid composition of wild-type *C. thermocellum* reported here is consistent with earlier studies (22), which likewise identified iso-branched fatty acids, specifically iso-16:0 and iso-18:0, as predominant fatty acid species, with lower levels of the corresponding straight-chain n-16:0 and n-18:0. One minor difference is that the prior study did not detect anteiso-17:0, likely due to its relatively low abundance. This and other minor discrepancies may reflect differences in growth conditions, as the earlier work employed rich complex medium rather than the minimal medium used in this study.

The physiological consequences of shifts in membrane lipid composition induced by exogenous alcohols and organic acids remain uncharacterized. These alterations could affect membrane fluidity, permeability, and integrity, which, in turn, may influence solvent tolerance, nutrient transport, and energy transduction. For example, enrichment of iso-branched fatty acids typically increases membrane rigidity and may reduce passive diffusion of small molecules, potentially mitigating alcohol-induced stress, whereas higher proportions of straight-chain fatty acids can enhance membrane fluidity but may also increase susceptibility to leakage (37–39). In support of this idea, preliminary data show that the Δ*pfor4* strain, which is unable to synthesize branched-chain fatty acids, exhibits elevated membrane permeability, as evidenced by increased ATP leakage during ethanol exposure compared to the wild-type (Fig. S5; Table S19). Changes in membrane composition could additionally impact the activity of membrane-bound enzymes, electron transport complexes, and transporters. Future studies are needed to determine how these remodeling events influence cellular physiology, solvent tolerance, and biofuel production in *C. thermocellum*.

Current ethanologenic strains of *C. thermocellum*, such as LL1570 (Table S16), have been engineered by deleting components of the native ethanol fermentation pathway, including Pfor isoenzymes (such as Pfor4) and the bifunctional dehydrogenase AdhE, and replacing them with heterologous enzymes from thermophilic ethanologenic organisms such as *T. saccharolyticum*. However, the absence of branched-chain fatty acids in the membrane lipids of LL1570 and similarly engineered strains may compromise membrane integrity and function, potentially contributing to their ethanol sensitivity (36). Restoring branched-chain fatty acid biosynthesis could therefore represent a strategy to enhance ethanol tolerance and support the development of more robust *C. thermocellum* strains capable of achieving higher ethanol titers under industrial fermentation conditions.

## MATERIALS AND METHODS

### Growth parameters

*C. thermocellum* (also known as *Hungateiclostridium thermocellum*, or *Ruminiclostridium thermocellum*) strains (DSM1313, LL1436, LL1437, LL1438, LL1563, LL1564, LL1567, LL1568, LL1569, and LL1570, see Table S16 for strain information) were revived from single-use anaerobic freezer stocks into thermophilic clostridia media (MTC) (14.6 mM [5 g/L] cellobiose, 40.2 mM MOPS, 0.0002% rezasurin, 6.17 mM potassium citrate, 6.19 mM citric acid, 7.04 mM Na_2_SO_4_, 7.35 mM KH_2_PO_4_, 29.8 mM NaHCO_3_, 33.3 mM [2 g/L] urea, 4.92 mM MgCl_2_, 1.36 mM CaCl_2_, 0.503 mM FeCl_2_, 5.69 mM L-cysteine HCl, Vitamin mix: [83.0 μM pyridoxamine HCl, 29.2 μM para-aminobenzoic acid, 8.19 μM biotin, 1.48 μM cobalamin, 11.9 μM thiamine], Trace metals: [2.53 nM Mn_2_Cl_2_, 2.10 nM CoCl_2_, 1.47 nM ZnCl_2_, 0.587 nM Cu_2_Cl_2_, 1.62 nM H_3_BO_3_, 0.413 nM Na_2_MoO_4_, 0.421 nM NiCl_2_]), and passaged in MTC for all relevant experiments. All growth was performed anaerobically in Hungate tubes at 55°C. For all cultures, passaging achieved at least a 1:20 dilution at all steps. Growth was assessed by optical density at 600_nm_ using a Thermo Scientific Genesys 50 UV-visible spectrophotometer. *C. thermocellum* strain (LL1111, LL1041, LL1210, see Table S16 for strain information) was revived from single-use anaerobic freezer stocks into CTFUD media 5 g/L Cellobiose, 10.44 g/L MOPS, 0.00002% rezasurin, 7.5 g/L sodium citrate tribasic dihydrate, 3.75 g/L potassium phosphate monobasic, 6.5 g/L ammonium sulfate, 13 g/L magnesium chloride hexahydrate, 0.65 g/L calcium chloride dihydrate, 0.005 g/L ferrous sulfate heptahydrate, 5 g/L HCl monohydrate, and 4.5 g/L yeast extract. Isotopically labeled alcohols and small organic acids were added to the media prior to culture growth at the following concentrations: ^13^C_1_-ethanol (5 g/L), ^2^H_9_-butanol (1.5 g/L), ^2^H_9_-isobutanol (3 g/L), and ^13^C_1_-sodium-butyrate (1.5 g/L). Concentrations were selected to induce a distinct phenotypic response while minimizing impact on growth rate based on preliminary data. Ethanol exposure resulted in a concentration-dependent increase in the levels of straight-chain fatty acids n-16:0 and n-18:0. Butanol exposure induced a marked shift in fatty acid composition, replacing the predominant iso-branched fatty acids iso-16:0 and iso-18:0 with their straight-chain counterparts n-16:0 and n-18:0. Isobutanol exposure, however, significantly elevated the levels of iso-16:0 and iso-18:0 while concurrently reducing the abundance of n-16:0 and n-18:0.

### Metabolite extraction

Working cultures were inoculated anaerobically with a 1:20 or greater dilution from overnight anaerobic cultures containing the same carbon source to an initial OD_600_ of 0.05. Cells were grown to mid-exponential phase (OD_600_ of 0.4–0.5), and then intracellular metabolites were collected inside the anaerobic chamber by vacuum filtration of 5–10 mL of culture through 3 μm hydrophilic nylon filters to separate cells from the media. Filters were then placed cell-side down in 1.5 mL of extraction solvent (40% acetonitrile, 40% methanol, and 20% water) and kept on metal blocks from the −80°C freezer in place of dry ice to quench metabolism and extract metabolites. Cells were washed off the filter using the solvent, which was collected, vortexed, and then centrifuged for 5 min at 4°C to remove cellular debris, and the supernatant was collected for LC-MS analysis.

### Lipid extraction

Cultures were grown to mid-log phase (~0.45 OD_600_), and 5 mL of culture was aliquoted into 15 mL glass culture tubes. The glass tubes with culture were centrifuged at 4,000 rpm at 4°C, and supernatant was removed, and cultures were flash frozen using a mixture of 190 proof ethanol and dry ice. Culture pellets were stored at −80°C until use. Frozen pellets were completely defrosted and kept on ice. Next, 300 μL of a butanol/methanol solution in a 3:1 (vol/vol) ratio was gently introduced using glass pipettes. Following this, the solution was vigorously vortexed for 30 s and subjected to further mixing with a foam tube holder for 20 min. 300 μL of a hexane/ethyl acetate solution in a 3:1 (vol/vol) ratio was added, vortexed for 30 s, and mixed for 20 min. The reaction was concluded by the addition of 300 µL of a 1% acetic acid solution. After vortexing for 30 s and mixing for 20 min, the solution was centrifuged at 4°C for 10 min at 4,000 rpm. 200 µL of the upper layer was collected for lipid analysis and fatty acid saponification and stored in 2 mL glass tubes. The collected layer was then dried under a stream of nitrogen gas. The resulting dried lipids were reconstituted in 55 µL of methanol, with 45 µL being used for LC-MS analysis.

### Fatty acid saponification

2 mL glass tubes containing 200 µL of extractant from lipid extraction were incubated at 80°C for 1 h. The extractant was then cooled briefly, and 100 µL of HPLC-grade formic acid was added. Subsequently, 900 µL of HPLC-grade hexane was added, and the extract was vortexed for 30 s and mixed for 20 min. Next, the extractant was centrifuged for 10 min at 4°C. 600 µL of the upper layer of the extract was aliquoted to new 2 mL glass tubes and dried down under nitrogen gas. 100 µL of acetonitrile, isopropanol, and water in a 65:30:5 (vol/vol) ratio was added to each tube and mixed for 30 s. The solution was then centrifuged for 10 min at 4°C. 80 µL of the solution was taken and used for LC-MS analysis.

### LC-MS metabolomics

Metabolomics LC-MS analyses were conducted using a Vanquish ultra-high-performance liquid chromatography (UHPLC) system (Thermo Scientific) coupled to a hybrid quadrupole-Orbitrap mass spectrometer (Q Exactive; Thermo Scientific) equipped with electrospray ionization operating in negative-ion mode as defined by Jacobson et al. (10). The chromatography was performed at 25°C using a 2.1 × 100 mm reverse-phase C_18_ column with a 1.7 μm particle size (Water; Acquity UHPLC BEH). Two chromatography solvents were used. The first used Solvent A (97:3 H_2_O: methanol + 10 mM tributylamine) and Solvent B (100% methanol) as follows: 0–2.5 min, 5% B; 2.5–17 min, linear gradient from 5% B to 95% B; 17–19.5 min, 95% B; 19.5–20 min, linear gradient from 95% B to 5% B; 20–25 min, 5% B. The second also used Solvent A and Solvent B (100% methanol) and was as follows: 0–2.5 min, 5% B; 2.5–7.5 min, linear gradient from 5% B to 20% B; 7.5–13 min, 20% B to 55% B; 13–18.5min, 55% B to 95% B; 18.5–19 min linear gradient from 95% B to 5% B; 19–25 min, 5% B. The flow rate was held constant at 0.2 mL/min for both chromatograph methods. Higher resolution (140,000 FWHM) was used for all dual-tracer experiments. Metabolites of interest were identified by retention times (based on pure standards) and monoisotopic mass using MAVEN (40) and El-MAVEN (41) software. Correction for the natural abundance of isotopes was completed using isocor (42).

### LC-MS lipidomics for fatty acids

The LC-MS lipidomics for fatty acid analysis was performed using a Vanquish UHPLC system (Thermo Scientific) coupled to a hybrid quadrupole Orbitrap mass spectrometer (Q Exactive plus; Thermo Scientific). The chromatography was done using a reverse-phase C18 column (Acquity UPLC CSH) (1.7-µm particle size, 2.1-by-100-mm column). Two chromatography solvents and a single gradient were used. The solvent compositions were as follows: Solvent A (60:40 Acetonitrile: H_2_O + 10 mM ammonium acetate + 250 µL/L acetic acid) and Solvent B (90:10 Isopropanol: Acetonitrile + 10 mM ammonium acetate + 250 µL/L acetic acid). The total run time was 33 min. Flow rate was held constant at 0.3 mL/min. The chromatography gradient was as follows: 2% solvent B until minute 1, linear increase to 100% solvent B until minute 20, held at 100% solvent B until minute 27, linear decrease to 2% solvent B until minute 28, and held at 2% solvent B until minute 33. Mass spectrometry analysis was conducted at negative ionization and began at minute 0 and ended at minute 27, after which the sample flow was directed to waste. Correction for the natural abundance of isotopes was completed using isocor (42).

### LC-MS/MS lipidomics for lipids

The LC-MS lipidomics for lipid analysis was performed using a Vanquish UHPLC system (Thermo Scientific) coupled to a hybrid quadrupole Orbitrap mass spectrometer (Q Exactive plus; Thermo Scientific). The chromatography was done using a reverse-phase C18 column (Acquity UPLC CSH) (1.7-µm particle size, 2.1-by-100-mm column). Two chromatography solvents and a single gradient were used. The solvent compositions were as follows: Solvent A (70:30 Acetonitrile: H_2_O + 10 mM ammonium acetate + 250 µL/L acetic acid) and Solvent B (90:10 isopropanol:acetonitrile + 10 mM ammonium acetate + 250 µL/L acetic acid). The total run time was 35 min. Flow rate was held constant at 0.3 mL/min. The chromatography gradient was as follows: 2% solvent B until minute 2, linear increase to 30% solvent B until minute 5, linear increase to 99% solvent B until minute 22, held at 99% solvent B until minute 29, linear decrease to 2% solvent B until minute 30, held at 2% solvent B until end of run at minute 35. Mass spectrometry analysis was conducted in both negative and positive ionization and began at minute 1 and ended at minute 29, after which the sample flow was directed to waste. Correction for the natural abundance of isotopes was completed using isocor (42).

## Data Availability

All data will be made available on request. Bacterial strains will be made available upon request.

## SUPPLEMENTAL MATERIAL

Fig. S1 msystems.01345-25-s0001.eps Effect of alcohol and acid exposure on membrane lipid composition in the Δ*adhE* strain.

Fig. S2 msystems.01345-25-s0002.eps AdhE is required for incorporation of exogenous alcohols into membrane lipids.

Fig. S3 msystems.01345-25-s0003.eps Acyl chain composition of membrane lipids in the Δ*pfor4* strain.

Fig. S4 msystems.01345-25-s0004.eps Canonical branched-chain fatty acid synthesis via Bkd.

Fig. S5 msystems.01345-25-s0005.eps Increased ATP leakage in the Δp*for4* strain under ethanol stress.

Supplemental Tables msystems.01345-25-s0006.xlsx Tables S1 to S19.

ASM does not own the copyrights to Supplemental Material that may be linked to, or accessed through, an article. The authors have granted ASM a non-exclusive, world-wide license to publish the Supplemental Material files. Please contact the corresponding author directly for reuse.
